# Efficacy of paclitaxel, carboplatin, and bevacizumab for cervical cancer: A protocol for systematic review and meta-analysis

**DOI:** 10.1097/MD.0000000000020558

**Published:** 2020-06-12

**Authors:** Li Zhang, Chun-yan Zheng, Jin-hui Cao, Shu-ling Luo

**Affiliations:** a Department of Gynecology; b Department of Gynecology and Obstetrics, 4th (Xing Yuan) Hospital of Yulin, Yulin, Shaanxi, China.

**Keywords:** bevacizumab, carboplatin, cervical cancer, paclitaxel

## Abstract

**Background:**

Cervical cancer (CC) is a very common and malignant tumor in female population. Although a variety of single medications are reported to treat this condition, they all have limited efficacy. Previous studies have reported the combination of paclitaxel, carboplatin, and bevacizumab (PCB) can be used for the treatment of patients with CC effectively. However, no systematic review has explored its efficacy and safety. This study will address its efficacy and safety systematically and comprehensively.

**Methods:**

The following electronic databases will be retrieved from their inceptions to the January 1, 2020 to identify all potential associated studies: MEDLINE, EMBASE, Cochrane Library, Scopus, Web of Science, CINAHL, Google scholar, and Chinese Biomedical Literature Database. We will include randomized controlled trials (RCTs) of adult women (≥18 years) with CC globally. Eligible interventions will target any forms of PCB. The study methodological quality of all included studies will be appraised using Cochrane risk of bias tool. Statistical analysis will be undertaken using RevMan 5.3 software. In addition, we will perform a narrative synthesis to describe quality and content of the evidence.

**Results:**

This study will summarize recent evidence and provide quality evidence for the efficacy and safety of PCB on CC.

**Conclusion:**

The findings of this study will seek to identify the efficacy and safety of PCB and suggest future directions for research efforts targeting CC among this population.

**Systematic review registration:**

INPLASY202040195.

## 1 Introduction

Cervical cancer (CC) is one of the most common cancers as a pertinent global health issue in the women population,^[1–3]^ which is associated with a huge number of deaths each year.^[4–6]^ It is estimated that there are 569,800 new cases annually with 311,400 deaths worldwide in 2018.^[7]^ Although a variety of single chemotherapy medications are reported to treat CC, there is still insufficient efficacy for the treatment of CC.^[8–12]^ Thus, the combination of single drug in treating CC is very necessary. Studies have shown that combination of paclitaxel, carboplatin, and bevacizumab (PCB) have utilized for the treatment of patients with CC.^[13–21]^ However, no systematic review on this topic has been conducted. Thus, we believe this systematic review will provide evidence on the efficacy and safety of PCB for the treatment of CC.

## 2 Methods

### 2.1 Study registration

We have registered this study on INPLASY202040195. It is being reported based on the Preferred Reporting Items for Systematic Reviews and Meta-Analysis Protocol statement guidelines.^[22]^

### 2.2 Ethics and dissemination

It is not necessary to provide ethical approval in this study, because we only analyze previous published data. The results of this study will be disseminated through a peer-reviewed journal or a conference meeting.

### 2.3 Study eligibility criteria

#### 2.3.1 Types of studies

We only include randomized controlled trials (RCTs) regardless their language and publication status. We will exclude any other studies, such as review, observational studies, and case studies.

#### 2.3.2 Types of participants

We will only consider female adults (≥18 years old) who were diagnosed as CC regardless their race, educational background, and economic status.

#### 2.3.3 Types of interventions

##### 2.3.3.1 Experimental interventions

We will include any types of PCB as a solely intervention for patients with CC. Any combination of PCB and others will be excluded.

##### 2.3.3.2 Control interventions

As a control management, it can be any therapies, but not the PCB.

#### 2.3.4 Type of outcome measurements

##### 2.3.4.1 Primary outcomes

1. Overall survival (defined as the time from randomization to death from any cause); and
2. Progression-free survival (defined as the time from randomization until first evidence of tumor progression or until death from any cause).

##### 2.3.4.2 Secondary outcomes

1. Recurrence-free survival;
2. Disease-free survival;
3. Quality of life, as measured by 36-Item Short Form Health Survey or other related tools; and
4. Toxicities.

### 2.4 Search strategy and data management

#### 2.4.1 Search strategy

The following electronic databases will be retrieved by professional librarians for all potential studies from their inceptions onwards to the January 1, 2020: MEDLINE, EMBASE, Cochrane Library, Scopus, Web of Science, CINAHL, Google scholar, and Chinese Biomedical Literature Database. No limitations of language and publication status will be applied to the search process. We will only consider RCTs of PCB for the treatment of adult females (≥18 years) with CC. A detailed description of search strategy for Cochrane Library is made in Table 1. Similar search strategies for other electronic databases will be adapted. We will also examine conference proceedings and reference lists of all included studies to avoid losing any eligible studies.

**Table 1:**
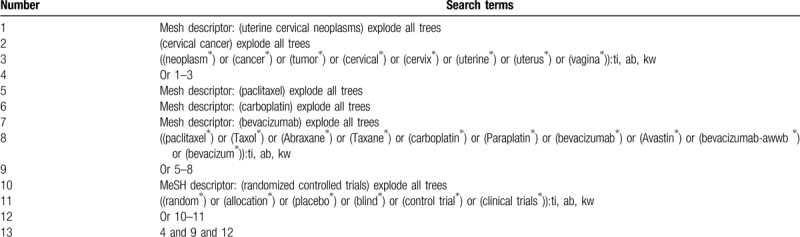
Search strategy used in Cochrane Library database.

#### 2.4.2 Study selection

After excluding duplicates, the titles and abstracts of all remaining studies will be checked carefully, and irrelevant literatures will be removed. After that, the rest articles will be read in full-text against all eligibility criteria. Two independent authors will carry out all procedures of study selection. Discrepancies at all screening steps between 2 authors will be figured out by a third author and a final decision will be made. We will record and show all study selection in a flow diagram.

#### 2.4.3 Data extraction and management

Extracted data from the included articles will be collected by 2 independent authors using a standardized template sheet developed specifically for this study. A third author will help to solve any discrepancies between 2 authors. Data to be collected from included articles are as follows:

1. First author and year of publication;
2. Subject population (age, diagnostic criteria);
3. Study design (details of randomization, blind, concealment and allocation);
4. Recruitment variables (trial setting, strategy, eligibility criteria);
5. Intervention and comparator details (delivery modes, delivery methods, dosage, frequency, duration);
6. Study outcome information (primary and secondary outcome measurements); and
7. Funding information.

#### 2.4.4 Dealing with missing data

If we find any missing or unclear information, we will contact primary authors to request them. If it is not available, we will only analyze data at hand.

### 2.5 Study quality assessment

Study quality in all trials will be independently evaluated using Cochrane risk of bias tool by 2 authors. It will be used for study quality assessment through 7 aspects. Each one is further graded as high, unclear or low risk of bias in this study. Any different opinions will be solved by a third author through discussion.

### 2.6 Strategy for statistical analysis

RevMan 5.3 software will be employed for statistical analysis in this study.

#### 2.6.1 Measurement of treatment effect

We will exert dichotomous outcome data using risk ratio and 95% confidence intervals (CIs), and continuous outcome data using mean difference or standardized mean difference and 95% CIs.

#### 2.6.2 Data synthesis

Heterogeneity among included trials will be determined by *I*^2^ test. If *I*^2^ ≤ 50%, it indicates low level of heterogeneity and a fixed-effect model will be used. We will plan to carry out a meta-analysis if sufficient data are collected on the same outcome measurement. If *I*^2^ > 50%, it means high level of heterogeneity, and a random-effect model will be utilized. At the same time, we will undertake subgroup analysis to find out if there are some possible factors that are responsible for the high level of heterogeneity. Additionally, a narrative synthesis of eligible trials will be conducted. It will include qualitatively summarizing all collected data. Quantitative summaries of extracted data will be reported, such as overall survival, and progression-free survival.

#### 2.6.3 Subgroup analysis

Subgroup analysis will be undertaken according to the different characteristics of study, patient information, study methods, intervention and comparators, and outcomes.

#### 2.6.4 Sensitivity analysis

Sensitivity analysis will be performed to explore the stability of integrated outcome data by removing trials with high risk of bias.

#### 2.6.5 Reporting bias

We will use funnel plot and Egger regression test to identify if there are any reporting bias when sufficient trials are included (normally more than 10 trials).

## 3 Discussion

This study is the first one to yield high quality evidence on the efficacy and safety of PCB for the treatment of patients with CC. This information will be helpful to notify the development of PCB for this population. Potential limitations in this study may consist of a restricted number of eligible trials, trials with small sample sizes, insufficient essential information of eligibility, interventions, controls or outcome measurements. Findings from this study may provide solid data and robust evidence of PCB for CC either for the clinician and patients or for further researchers and health policy makers.

## Author contributions

**Conceptualization:** Li Zhang, Chun-yan Zheng, Jin-hui Cao, Shu-ling Luo.

**Data curation:** Li Zhang, Jin-hui Cao.

**Formal analysis:** Chun-yan Zheng, Shu-ling Luo.

**Investigation:** Shu-ling Luo.

**Methodology:** Li Zhang, Jin-hui Cao.

**Project administration:** Shu-ling Luo.

**Resources:** Li Zhang, Chun-yan Zheng, Jin-hui Cao.

**Software:** Chun-yan Zheng, Jin-hui Cao.

**Supervision:** Shu-ling Luo.

**Validation:** Li Zhang, Chun-yan Zheng, Jin-hui Cao, Shu-ling Luo.

**Visualization:** Li Zhang, Chun-yan Zheng, Jin-hui Cao, Shu-ling Luo.

**Writing – original draft:** Li Zhang, Chun-yan Zheng, Jin-hui Cao, Shu-ling Luo.

**Writing – review & editing:** Li Zhang, Chun-yan Zheng, Shu-ling Luo.
